# Relationship between first pass effect NLR and PLR in mechanical thrombectomy for acute anterior circulation large-vessel occlusion

**DOI:** 10.3389/fneur.2025.1490127

**Published:** 2025-07-02

**Authors:** Guozhang Lu, Peijian Wang, Bin Xv, Hang Lv, Liyong Zhang, Jiyue Wang, Jiheng Hao

**Affiliations:** ^1^Department of Neurosurgery, Liaocheng People’s Hospital, Liaocheng, China; ^2^School of Clinical Medicine, Weifang Medical University, Weifang, China; ^3^Department of Neurosurgery, XD Group Hospital, Xian, China

**Keywords:** mechanical thrombectomy, first pass effect, neutrophil-lymphocyte ratio, platelet-lymphocyte ratio, acute anterior circulation large-vessel occlusion

## Abstract

**Purpose:**

The study investigated the correlation between the neutrophil-lymphocyte ratio (NLR) and the platelet-lymphocyte ratio (PLR) concerning the first-pass effect (FPE) observed during mechanical thrombectomy subsequent to acute ischemic stroke (AIS).

**Methods:**

Patients diagnosed with AIS in the anterior circulation, who underwent mechanical thrombectomy between January 2020 and December 2022, were assessed. Various data were collected, including blood cell counts, general information, relevant surgical and clinical details, and functional outcomes determined by the Modified Rankin Scale (MRS) score ≤2 at 3 months. Logistic regression was utilized to identify independent factors predicting the first-pass effect (FPE) and to explore the associations between FPE and the NLR and PLR. Critical NLR and PLR values were examined using Receiver-operating characteristics (ROC) curves.

**Results:**

A total of 233 patients were enrolled and categorized into either the FPE or MPE groups based on the success of the initial thrombectomy. The FPE group showed significant distinctions compared to the MPE group in both NLR and PLR levels: NLR (3.63 vs. 4.90, *p* < 0.001), PLR (134.92 vs. 164.77, *p* = 0.001). Both univariate and multivariate regression analyses demonstrated the independent predictive ability of NLR and PLR for assessing the risk of FPE during mechanical thrombectomy, with NLR (Adjusted Odds ratio (OR) 0.764; 95% CI 0.665–0.878, *p* = 0.001) and PLR (Adjusted OR0.993; 95% CI 0.989–0.998, *p* = 0.002). Moreover, the ROC curves delineated critical threshold values of 4.34 and 148.03 for NLR and PLR, respectively.

**Conclusion:**

The increase of NLR and PLR may be related to the failure of FPE.

## Introduction

Acute ischemic stroke (AIS) represents approximately 86% of stroke cases and stands as one of the most prevalent, incapacitating, and life-threatening conditions (1). Prompt restoration of the obstructed blood vessels is crucial for salvaging the ischemic penumbra. Studies report that in cases of AIS accompanied by large-vessel occlusion, the use of Endovascular therapy (EVT) is effective in enhancing functional outcomes compared to medical therapy, without elevating the risk of symptomatic intracranial hemorrhage (SICH) (2). Nevertheless, despite reductions in mortality and improvements in functional outcomes, over 60% of AIS patients still experience adverse outcomes. This underscores the need for further research and strategies to enhance the effectiveness of stroke treatments (2–4).

The concept of FPE was first introduced in an analysis by Zaidat et al., with the study showing a 90-day favorable prognosis rate of 61.3%, a 90-day mortality rate of 16.3%, and a distal embolization rate of 5.7% in the FPE group, demonstrating a significant correlation between FPE and a favorable prognosis (5). In a meta-analysis, patients in the FPE group showed better outcomes and lower mortality rates compared to those in the MPE group (6). A prospective, multicenter study showed that the functional independence rate (MRS ≤ 2) for patients in the FPE group was 52.7%, with a good recovery rate (MRS ≤ 1) of 49% (7). Prolonged surgery time and increased number of thrombectomies may lead to endothelial damage, reocclusion of vessels, or thrombus migration causing distal occlusion, thus reducing the effectiveness of mechanical thrombectomy and patient prognosis (8–11).

The inflammatory response is widely recognized as closely associated with AIS development and progression (12). During AIS, neutrophils can infiltrate ischemic brain tissue by breaching the blood–brain barrier, thereby inducing further damage to brain tissue through the release of inflammatory factors. This process can exacerbate the injury caused by the stroke (13). Platelets play a critical role in the blood clotting process. Under the stimulation of inflammatory mediators, they release additional procoagulant factors, intensifying aggregation activity and escalating thrombus burden. This heightened thrombotic activity significantly contributes to AIS pathophysiology (14). Previous studies have shown a link between NLR and functional prognosis 3 months after AIS (15, 16). Limited information exists regarding the connections between the NLR and the platelet-lymphocyte ratio (PLR) concerning the attainment of the First-Pass Effect (FPE) in cases involving anterior circulation large-vessel occlusion undergoing mechanical thrombectomy. Hence, our study aims to explore the correlation between PLR and NLR and FPE, focusing on assessing the predictive significance of PLR and NLR in achieving FPE during mechanical thrombectomy.

Its objective was to examine the association between the First-Pass Effect (FPE) during mechanical thrombectomy and NLR and PLR.

## Methods

### Patients

Patients with AIS who underwent mechanical thrombectomy in the Department of cerebrovascular Neurosurgery in Liaocheng People’s Hospital from January 2020 to December 2022 were retrospectively analyzed. The time for blood sample collection was immediately upon the patient’s admission to the emergency department, taking venous blood. Our study was approved by the Ethics Committee of our hospital and exempted from signing informed consent. The following criteria were utilized for final enrollment.

The inclusion criteria comprised: 1. Age > 18; 2. Absence of intracranial hemorrhagic lesions; 3. Confirmation of anterior circulation large-vessel occlusion through digital subtraction angiography; 4. Receipt of endovascular treatment; 5. Onset of symptoms within less than 6 h or between 6 and 24 h, while meeting the DEFUSE-3 or DAWN trial selection criteria.

The exclusion criteria in our study included: 1. Absence of NLR and PLR values; 2. Incomplete clinical data or follow-up information; 3. Confirmed posterior circulation pathology identified by DSA; 4. Confirmed anterior circulation tandem lesions identified by DSA; 5. Patients diagnosed with pre-existing active infections, rheumatic immune disorders, or tumors.

“FPE” was defined as follows: 1. The thrombectomy device achieves single-pass or initial large-vessel occlusion and downstream reperfusion (Modified treatment in cerebral infarction, mTICI 2b-3); 2. The absence of rescue measures such as balloon angioplasty, stent placement, intra-arterial thrombolysis, or the use of alternative catheters.

The definition of ‘MPE’ in this study encompasses the following criteria: Reperfusion is achieved by employing the thrombectomy device multiple times or through the utilization of rescue measures (mTICI 2b-3).

### Endovascular treatment

The surgery was performed under resting compound general anesthesia. Prior to thrombectomy, routine aortic arch and whole cerebral angiography were conducted to identify the occlusion site and assess collateral circulation compensation. The thrombectomy techniques employed at our center primarily include: simple thrombus aspiration technique, stent retrieval technique alone, and combined stent-retrieval with aspiration technique. The operator selects the appropriate surgical approach based on thrombus characteristics and occlusion location. Each insertion and withdrawal of different thrombectomy devices is counted as one maneuver. If inadequate perfusion occurs due to localized vascular stenosis following vascular recanalization, balloon angioplasty or stent implantation is utilized.

### Baseline characteristics

The collected basic patient information encompassed demographic factors such as sex, age, stroke history, hypertension status, diabetes status, and atrial fibrillation. Additional data on hematology and inflammatory status collected immediately upon admission to the emergency department include measurements of white blood cells, platelets, neutrophils, and lymphocytes, and calculations of NLR and PLR. Severity of AIS upon admission was assessed utilizing the National Institutes of Health Stroke Scale (NIHSS) score in conjunction with the Alberta Stroke Program Early CT Score (ASPECTS) to evaluate ischemic brain damage via CT imaging. Surgical data encompassed details such as the site of occlusion, collateral circulation, number of thrombectomy passes, interval between symptom onset and puncture, and duration from puncture to reperfusion. Postoperative data included details on emboli escape, contrast agent leakage, intracranial hemorrhage (ICH), and the modified Rankin Scale (mRS) score assessed after 3 months.

Collateral circulation was assessed utilizing the American Society of Intervention and Therapeutic Neuroradiology/Society of Interventional Radiology (ASITN/SIR) grading system. Scores ranging from 2 to 3 indicated moderately good to good collateral circulation (17).

Vascular reperfusion assessment utilized the Modified Thrombolysis in Cerebral Infarction (mTICI) score, defining successful reperfusion as achieving mTICI 2b/3 (18). Favorable functional outcomes were represented as mRS scores between 0 and 2 (19), indicative of enhanced functional independence. Unfavorable functional outcomes were denoted by scores ranging from 3 to 6, where a score of 6 signified mortality.

### Statistical analysis

In our study, we employed statistical analysis using SPSS 27.0. For missing data, we use the direct deletion method. Normally distributed data are shown as mean ± standard deviation (x ± s) and were compared with t-tests. Non-normally distributed data are shown as medians and Mann–Whitney U tests were used. Categorical data are represented as counts and percentages [*n* (%)]. In the outcome event, the significant variables (*p* < 0.1) found in the univariate analysis were included in the multivariate binary logistic regression analysis to determine the independent factors affecting FPE. ROC curves were used for evaluating of predictive efficacy. *p* < 0.05 was considered statistically significant.

## Results

**Figure 1:**
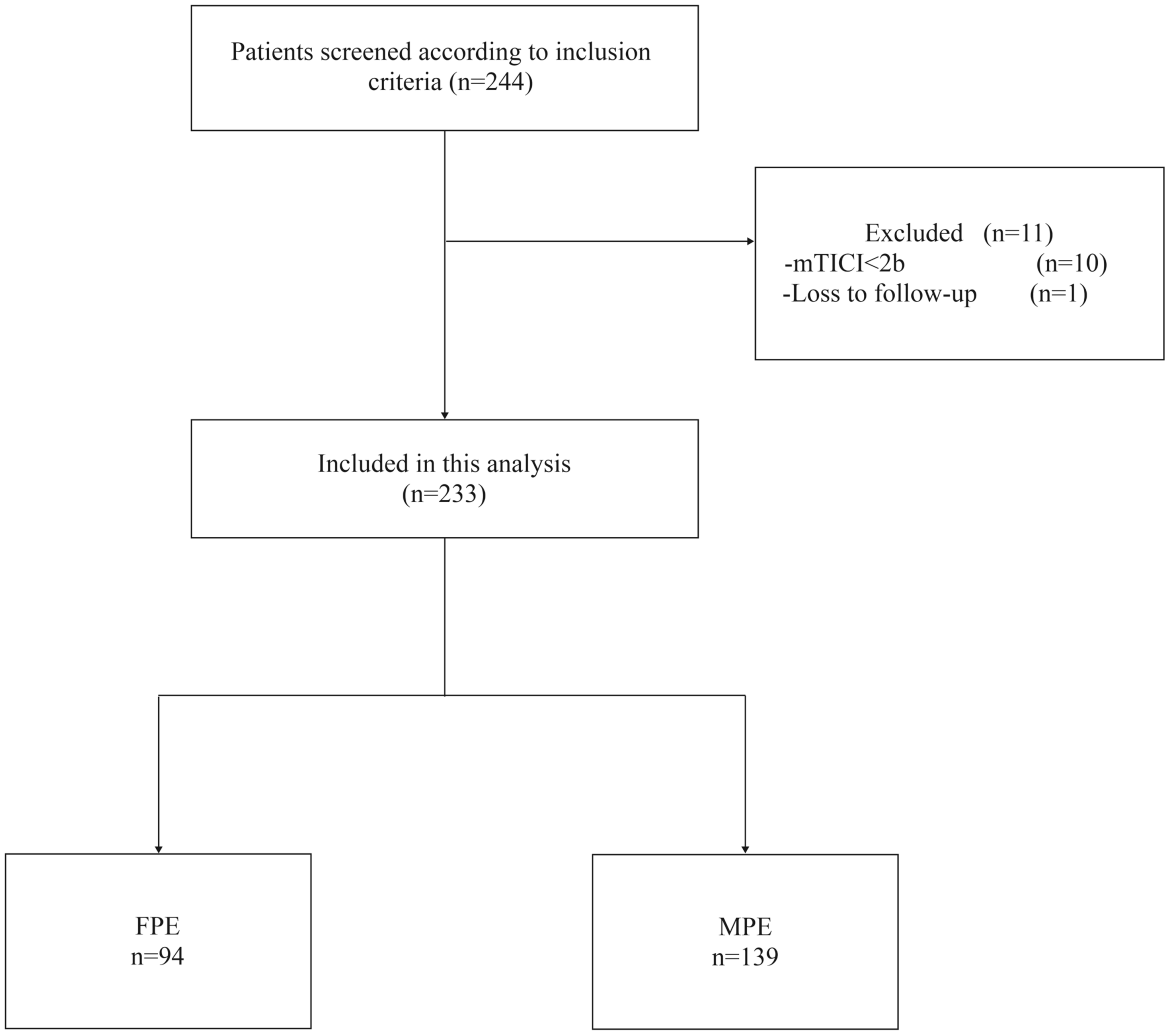
Flowchart of patient selection.

As shown in Figure 1, our study initially screened 244 patients. Among them, 10 patients were excluded due to the final mTICI < 2b, and 1 patient could not be followed up because the phone number could not be reached. Eventually, 233 patients were included, aged between 32 and 99 years, with an average age of 68.1 ± 11 years, of which 133 were male (57%). The FPE group comprised 94 patients, while the MPE group consisted of 139 patients. Baseline characteristics upon admission revealed higher levels of collateral circulation in FPE patients compared to MPE patients (ASITN/SIR 2–3, 66% vs. 34.5%, *p* < 0.001). The FPE group exhibited lower neutrophil counts (5.10 (3.69–6.37) vs. 6.24 (4.32–8.40), *p* < 0.001), increased lymphocyte counts (1.37 (1.09–1.84) vs. 1.21 (0.89–1.54), *p* = 0.003), and notably lower NLR (3.63 (2.46–5.00) vs. 4.90 (3.08–7.24), *p* < 0.001) and PLR (134.92 (105.34–173.47) vs. 164.77 (117.90–216.39), *p* = 0.001) compared to MPE patients (Table 1).

**Table 1:** Basic patient information and laboratory test results.

Basic information	FPE (*N* = 94)	MPE (*N* = 139)	*p*-value
Age (years)	69.6 ± 9.43	67 ± 11.8	0.076
Female/Male (*n*)	46/48	54/85	0.127
Risk factors, *n* (%)			
Smoking	46 (49)	71 (53)	0.519
Drinking	46 (49)	76 (55)	0.389
Previous stroke	16 (17)	20 (14)	0.585
Hypertension	71 (76)	89 (64)	0.063
Coronary artery disease	21 (22)	34 (25)	0.709
Atrial fibrillation	60 (64)	79 (57)	0.286
Diabetes	28 (30)	34 (25)	0.367
Hypercholesterolemia	10 (11)	25 (18)	0.124
Stroke cause, *n* (%)			<0.001
LAA	21 (22.3)	64 (46)	
CE	70 (74.5)	72 (51.8)	
Others	3 (3.2)	3 (2.2)	
ASITN/SIR, *n* (%)			<0.001
0–1	32 (34)	91 (65.5)	
2–3	62 (66)	48 (34.5)	
Laboratory data[M (Q1, Q3)]			
Leukocyte (×109/L)	7.06 (5.67–8.28)	7.39 (5.92–9.27)	0.184
Platelet (×109/L)	183 (157–219)	192 (164–228)	0.115
Neutrophils (×109/L)	5.10 (3.69–6.37)	6.24 (4.32–8.40)	<0.001
Lymphocyte (×109/L)	1.37 (1.09–1.84)	1.21 (0.89–1.54)	0.003
NLR	3.63 (2.46–5.00)	4.90 (3.08–7.24)	<0.001
PLR	134.92 (105.34–173.47)	164.77 (117.90–216.39)	0.001

FPE, first pass effect; MPE, multiple-pass effect; PLR, platelet-lymphocyte ratio; NLR, neutrophil-lymphocyte ratio; ASITN/SIR, the American Society of Interventional and Therapeutic Neuroradiology/Society of Interventional Radiology; LAA, large artery atherosclerosis; CE, Cardioembolism.

The interval femoral artery puncture and reperfusion was shorter in the FPE group (71 (50–82) vs. 89 (62–110), *p* < 0.001), and the admission ASPECT scores were higher (8 (7–9) vs. 7 (6–8), *p* < 0.001) (Table 2).

**Table 2:** Clinical data of patients.

Clinical data	FPE (*N* = 94)	MPE (*N* = 139)	*p*-value
Admission NIHSS score	18 (12–27)	18 (14–26)	0.335
ASPECT score at admission	8 (7–9)	7 (6–8)	<0.001
Intravenous tPA	58 (62)	74 (53)	0.201
Time from onset to puncture, min	292 (235–380)	296 (218–415)	0.874
PTR, min	71 (50–82)	89 (62–110)	<0.001
Occlusion position, *n* (%)			0.151
ICA	36 (38.3)	56 (40.3)	
MCA	58 (61.7)	83 (59.7)	

ASPECTS, the Alberta Stroke Program Early CT Score; ICA, internal carotid artery; MCA, middle cerebral artery; NIHSS, National Institute of Health Stroke Scale; PTR, puncture to recanalization; tPA, tissue plasminogen activator; FPE, first pass effect; MPE, multiple-pass effect.

Compared to patients in the MPE group, patients in the FPE group demonstrated a favorable prognosis at 90 days (MRS 0–2, 63.8% vs. 48.2%, *p* < 0.001), and the incidence of SICH postoperatively was higher in the MPE group (4.3% vs. 10.8%, *p* < 0.001) (Table 3).

**Table 3:** Clinical prognosis.

Clinical prognosis	FPE (*N* = 94)	MPE (*N* = 139)	*p*-value
SICH, *n* (%)	4 (4.3)	15 (10.8)	<0.001
MRS 0–2 at 90 days, *n* (%)	60 (63.8)	67 (48.2)	<0.001

sICH, symptomatic intracerebral hemorrhage; MRS, modified Rankin Scale; FPE, first pass effect; MPE, multiple-pass effect.

The study performed multiple-factor regression analyses to identify independent risk factors impacting FPE (Femoral Percutaneous Endovascular Therapy). In the multiple-factor regression analysis, after adjusting for various factors, the results indicated that NLR (OR 0.764; 95% CI 0.665–0.878, *p* = 0.001), PLR (OR 0.993; 95% CI 0.989–0.998, *p* = 0.002), were all independent factors influencing FPE. Both NLR and PLR were identified as independent predictors of FPE risk (Tables 4, 5).

**Table 4:** Multivariate logistic regression of association between NLR and FPE.

Clinical outcomes	Adjusted OR	95% CI	*p* value
NLR	0.764	0.665–0.878	0.001
Stroke cause	0.270	0.135–0.540	0.001
ASITN/SIR	4.608	2.433–8.727	0.001
ASPECT score at admission	1.399	1.084–1.806	0.010

OR, odds ratio; CI, confidence interval; NLR, neutrophil-lymphocyte ratio; ASPECTS, the Alberta Stroke Program Early CT Score; ASITN/SIR, the American Society of Interventional and Therapeutic Neuroradiology/Society of Interventional Radiology.

**Table 5:** Multivariate logistic regression of association between PLR and FPE.

Clinical outcomes	Adjusted OR	95% CI	*p* value
PLR	0.993	0.989–0.998	0.02
Stroke cause	0.278	0.142–0.545	0.001
ASITN/SIR	4.898	2.608–9.200	0.001
ASPECT score at admission	1.398	1.084–1.802	0.010

OR, odds ratio; CI, confidence interval; PLR, platelet-lymphocyte ratio; ASPECTS, the Alberta Stroke Program Early CT Score; ASITN/SIR, the American Society of Interventional and Therapeutic Neuroradiology/Society of Interventional Radiology.

**Figure 2:**
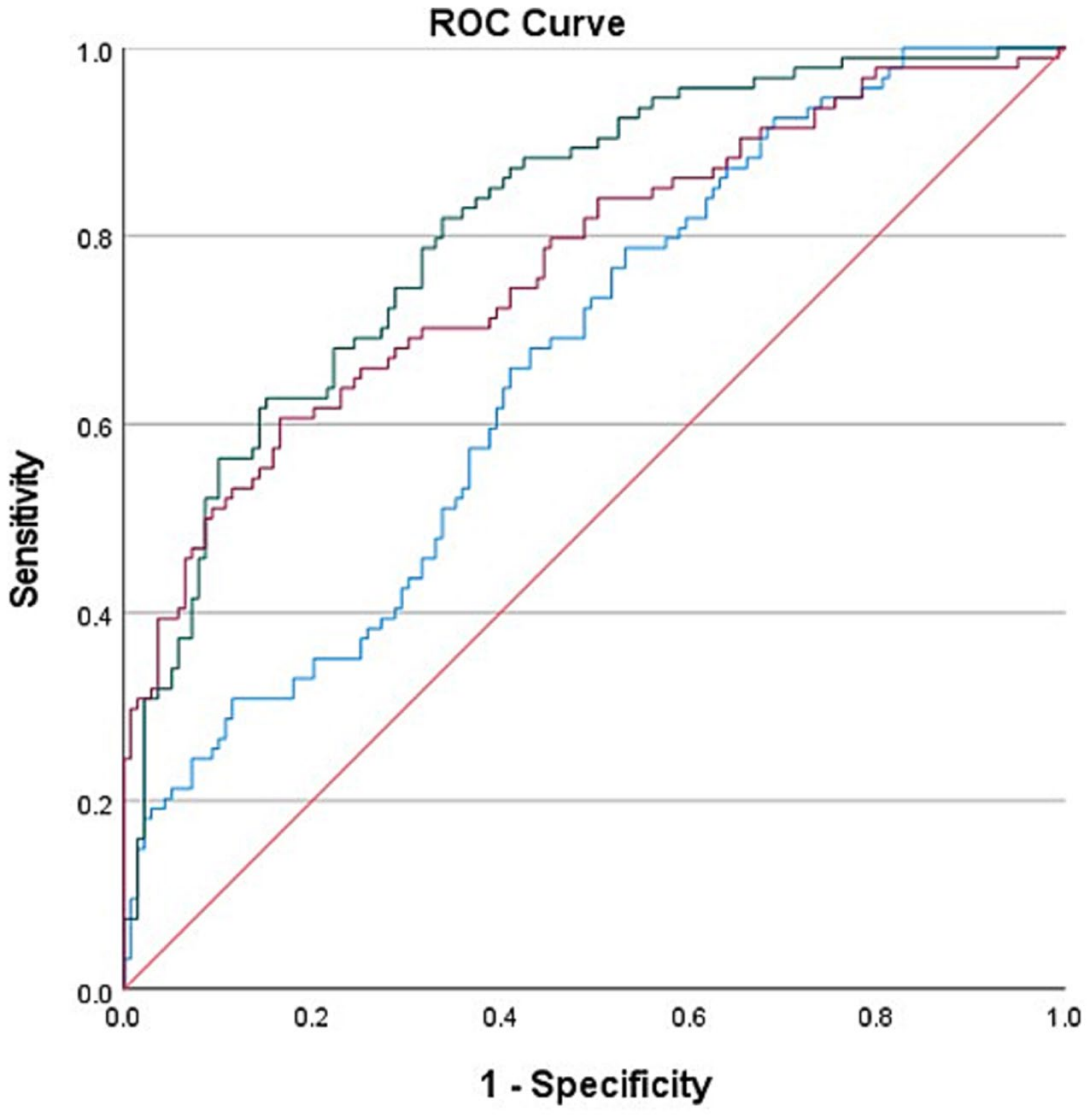
Use biological indicators (PLR and NLR), clinical indicators (PTR, ASPECT score at admission, Stroke cause, ASITN/SIR) and the combined ROC curve of the two indexes were used to evaluate the effect of FPE prediction. The area under the curve varies from 0.665 (95% Cl 0.595–0.734, *p* < 0.001) and 0.771 (95% Cl 0.708–0.834, *p* < 0.001) for biological indicators (blue) and clinical indicators (red) increased to 0.816 (95% Cl 0.761–0.870 *p* < 0.001) for joint indicators (green).

ROC curves based on biological markers (PLR and NLR), clinical indicators (PTR, ASPECT score at admission, Stroke cause, ASITN/SIR), and a combination of these indicators were used to evaluate the predictive effectiveness for FPE. In these ROC curves, biological markers are depicted in blue, clinical indicators in red, and the combined metrics in green. In our multifactorial combined prediction for FPE, the AUC increased from 0.665 (95% CI 0.595–0.734, *p* < 0.001) for biological indicators (blue) and 0.771 (95% CI 0.708–0.834, *p* < 0.001) for clinical indicators (red) to 0.816 (95% CI 0.761–0.870, *p* < 0.001) for joint indicators (green) (Table 6 and Figure 2).

**Table 6:** FPE was predicted by biological indicators, clinical indicators and Joint indicators.

Features	AUROC	95%CI	Cutoff value	Sensitivity (%)	Specificity (%)	*p* value
Biological indicators	0.665	0.595–0.734	0.255	78.7%	46.8	<0.001
Clinical indicators	0.771	0.708–0.834	0.441	60.6	83.5	<0.001
Joint indicators	0.816	0.761–0.870	0.481	81.9	66.2	<0.001

AUROC, Area under the curve; CI, confidence interval.

The determined cutoff value for NLR was 4.34 (sensitivity: 59.7%, specificity: 67%). PLR had a cutoff value of 148.03 (sensitivity: 54.7%, specificity: 76.2%).

## Discussion

This study aimed to explore the associations between NLR and PLR measured upon admission and FPE among patients experiencing acute large-vessel occlusion in the anterior circulation who underwent EVT. Findings from the study reveal an association between elevated NLR and PLR values and FPE failure, correlating these heightened values with adverse patient outcomes.

Numerous factors can impact the prognosis of mechanical thrombectomy. Successful reperfusion on the first attempt (FPE), when compared to multiple thrombectomy attempts, has the potential to enhance patient outcomes (6, 20). The unfavorable prognosis associated with multiple thrombectomy attempts might stem from vascular endothelial cell damage, thrombus escape, and a heightened risk of hemorrhagic transformation (21–23). Research has indicated that patients who achieve FPE during a medical procedure generally have a lower mortality rate, better prognosis, and a lower incidence of symptomatic intracranial hemorrhage (5). Thus, the clinical value of the FPE in mechanical thrombectomy is being promoted and explored.

The FPE concept was first proposed by the North American Solitaire Acute Stroke (NASA) registry (5). The mechanism through which FPE improves patient outcomes may involve several factors. Each pass of the thrombectomy device through a blood vessel can potentially cause vascular wall injury and distal embolization. Moreover, an increased number of thrombectomy attempts is linked to longer reperfusion times. Therefore, achieving FPE is beneficial (10, 17, 24–27).

Recent studies have shown the close involvement of inflammation in AIS development and progression, particularly in the early stages, with neutrophils being prominent inflammatory cells in the brain. These disrupt the blood–brain barrier through the release of proteases and other inflammatory agents, elevating the risk of postoperative sICH and leading to an enlargement of the infarcted brain area. Neutrophils also interact with platelets and coagulation factors, promoting thrombus formation, which increases the thrombus burden and poses challenges for vascular reperfusion (28–30). Lymphocyte subtypes can participate in various inflammatory responses, and they have the potential to reduce the infarcted area and improve functional deficits following AIS. However, the stress-associated release of corticosteroids promotes lymphocyte apoptosis, potentially leading to poorer early functional outcomes and prognosis (31, 32). Elevated NLR and PLR values are indicative a greater thrombus burden at the occlusion site, increased difficulty in achieving reperfusion, a higher likelihood of perioperative complications, and ultimately, a worse prognosis for the patient.

In our study, elevated NLR and PLR were linked with an increased likelihood of FPE failure (3.63 (2.46–5.00) vs. 4.90 (3.08–7.24), *p* < 0.001) and (134.92 (105.34–173.47) vs. 164.77 (117.90–216.39), *p* = 0.001). Furthermore, higher NLR and PLR were predictive of FPE failure (NLR (OR 0.764; 95% CI 0.665–0.878, *p* = 0.001)) and PLR (OR 0.993; 95% CI 0.989–0.998, *p* = 0.002). These findings are in agreement with those of Şengeze and Giray (33), who observed that increased NLR values were predictive of FPE failure in cases with acute middle cerebral artery occlusion following AIS and undergoing mechanical thrombectomy. However, the inclusion of occlusions in other anterior circulation vessels in this study adds to the persuasiveness of the results. The study by Orkun Sarioglu and colleagues found that PLR values were predictive of FPE (34). However, what sets this current study apart is that it excluded patients with preoperative active infections and those with a history of antibiotic use within 3 months. Additionally, patients with blood disorders, immune diseases, rheumatic diseases, malignancies, and those who used steroid medications were also excluded from the study.

As shown in Table 1, there was no statistically significant difference in lymphocyte counts between the two patient groups. This finding is consistent with existing reports in the literature (33). Furthermore, our linear regression analysis revealed variance inflation factor (VIF) values of 2.296 for both NLR and PLR, indicating acceptable collinearity levels. The low correlation between NLR and PLR suggests that their statistical independence was preserved in the analysis.

The reason may be Neutrophil Extracellular Traps (NETs) can limit microbial activity by promoting blood coagulation (35, 36). The interaction between NETs and platelets, as well as between activated platelets and neutrophils, creates a vicious cycle that leads to the formation of pathological thrombi (37). NETs have been detected in thrombi from patients with acute large vessel occlusion undergoing mechanical thrombectomy (38). Furthermore, researchers have pointed out that markers of NETs are associated with the severity of stroke (39–41). Therefore, in patients with acute large vessel occlusion, an increase in neutrophils and platelets, and consequently in NLR and PLR, suggests an increased thrombus burden, greater difficulty in reperfusion, a higher likelihood of perioperative complications, and ultimately poorer patient outcomes. Under normal physiological conditions, platelets do not adhere to the endothelium. However, the release of pro-inflammatory factors by endothelial cells and damage to the endothelium can lead to platelet aggregation and interaction with neutrophils, promoting the formation of atherosclerosis (42). Furthermore, the rupture of atherosclerotic plaques and the exposure of the lipid core can exacerbate platelet aggregation, leading to the formation of local thrombi (43). It has been discovered that platelets can identify and release a large number of cytokines and chemokines in response to microbial invasion (44, 45). These functions of platelets, in interaction with neutrophils, promote the formation of thrombi.

Our multifactorial combined analysis for predicting FPE showed higher AUC, sensitivity, and specificity than single-factor predictions, suggesting enhanced predictive capability (0.816 (95%CI 0.761–0.870 *p* < 0.001)). The NLR cutoff value was 4.34, while that for PLR was 148.03. The differences in the ROC areas and the NLR and PLR cutoff values between this study and the one conducted by Orkun Sarioglu and colleagues (with ROC areas of 0.730 and 0.847 and cutoff values of 3.22 and 126.3) could indeed be attributed to various factors (34), including potential differences in the study populations, such as racial or genetic variations. These variations in study populations can lead to differences in baseline NLR and PLR values, as well as the thresholds for predicting outcomes like FPE.

Mechtouff L et al.’s research found that lower admission levels of interleukin IL-6 are associated with FPE (OR 0.66, 95%CI 0.46–0.94), with a threshold value of 3.0 pg./mL (46). Research has shown a relationship between Syndecan-1 in arterial plasma and acute large vessel occlusion; during the acute phase of such occlusion, Syndecan-1 levels significantly increase, and begin to decrease after successful reperfusion (47). In our study, we found that NLR and PLR are independent risk factors for FPE. Future research could combine multiple factors from blood samples taken at different times to dynamically study their relationship with FPE. In previous studies, NLR has been associated with the prognosis of patients with traumatic brain injury (TBI) (48), similar value was found in patients with intracerebral hemorrhage (ICH) (49). In chronic diseases, NLR remains relevant, and these chronic conditions may be associated with the occurrence of AIS (50). Studies have found that diabetic patients have increased NLR ratios, especially those with poor blood sugar control. In diabetic AIS patients, a higher peripheral neutrophil count and a lower lymphocyte ratio lead to an increased NLR (51). Furthermore, in diabetic patients, PLR is significantly reduced in the pre-diabetic and early diabetic stages, but increases in the later stages (52). Obesity is considered to be associated with arteriosclerotic vascular diseases, and studies show that PLR is significantly elevated in obese patients (53). In infective endocarditis, there is also a higher risk of AIS and a higher baseline NLR, which can predict the prognosis.

In infective endocarditis, there is also a higher risk of AIS and a higher baseline NLR, which can predict the prognosis (54). Research has shown that NLR and PLR can be used to differentiate active rheumatoid arthritis (55). In cancer patients, NLR and PLR still hold diagnostic significance and can serve as blood biomarkers for early screening (56).

This study has several limitations. First, it is a single-center retrospective survey without prior intervention from researchers, which inevitably leads to bias in the results, and it is difficult to randomize the included patients, although we strictly implemented the corresponding inclusion and exclusion criteria, it is difficult to eliminate some factors that potentially affect NLR and PLR. Secondly, we only collected the results of blood cells in venous blood at the time of admission, and the choice of surgery by the surgeons based on their experience might have influenced the outcomes of FPE. In the future, a more comprehensive approach might include dynamic analysis of blood cell results from both venous and arterial blood and differentiating between various predictors and thrombectomy techniques, which could be more applicable to clinical practice discoveries and enhance the understanding of factors affecting thrombectomy outcomes after AIS.

In conclusion, in patients with acute anterior circulation large vessel occlusion, the increase of NLR and PLR may be related to the failure of FPE, and when combined with clinical indicators, it is even more related to the failure of FPE.

## Data Availability

The raw data supporting the conclusions of this article will be made available by the authors, without undue reservation.
